# Salivary miR-324-5p Is Associated with Periodontal Inflamed Surface Area in People Living with HIV Receiving Antiretroviral Therapy: A Case–Control Study

**DOI:** 10.3390/ijms27188117

**Published:** 2026-09-12

**Authors:** Mohammad Nurhamim, Aya Yokoi, Hideharu Hagiya, Takayuki Maruyama, Daisuke Ekuni

**Affiliations:** 1 Department of Preventive Dentistry, Graduate School of Medicine, Dentistry and Pharmaceutical Sciences, Okayama University, Okayama 700-8558, Japan; dr.nurhamim@gmail.com; 2 Department of Preventive Dentistry, Faculty of Medicine, Dentistry and Pharmaceutical Sciences, Okayama University, Okayama 700-8558, Japan; t-maru@md.okayama-u.ac.jp (T.M.); dekuni7@md.okayama-u.ac.jp (D.E.); 3 Department of Infectious Diseases, Graduate School of Medicine, Dentistry and Pharmaceutical Sciences, Okayama University, Okayama 700-8558, Japan; hagiya@okayama-u.ac.jp

**Keywords:** miR-324-5p, saliva, marker, HIV, periodontitis, extracellular vesicles, inflammation

## Abstract

Periodontitis is a common inflammatory disease that affects the tooth-supporting tissues. Salivary microRNA (miRNA) expression in extracellular vesicles (EVs) can reflect periodontal status. Both the prevalence and severity of periodontitis are higher in people with human immunodeficiency virus (PWH). This study aimed to identify miRNA in salivary EVs associated with periodontitis in PWH receiving antiretroviral therapy (ART). The case group comprised PWH with periodontitis undergoing ART, while the control group included individuals with periodontitis but without HIV infection. The study was conducted in two phases. In the first phase, a specific miRNA was identified in the case group using next-generation sequencing analysis, resulting in the selection of hsa-miR-324-5p as a candidate marker of periodontitis. In the second phase, the association between miRNA expression of hsa-miR-324-5p and periodontal status in the case group was evaluated using real-time-quantitative PCR (RT-qPCR). RT-qPCR analysis demonstrated that hsa-miR-324-5p expression was significantly associated with periodontal inflamed surface area (PISA). Bioinformatic analysis identified 10 potential hub genes for hsa-miR-324-5p that are involved in protein kinase binding and are associated with the adherens junction pathway, that may be contributing to the pathogenesis of periodontitis. Salivary hsa-miR-324-5p derived from EVs may be associated with the severity of periodontitis among PWH receiving ART.

## 1. Introduction

miRNAs are a class of small non-coding RNAs, typically 19–25 nucleotides in length, that regulate gene expression at the post-transcriptional level by inhibiting translation or promoting mRNA degradation [1]. miRNAs can be selectively packaged into extracellular vesicles (EVs) and are detectable in various body fluids, including blood, urine, saliva, and cerebrospinal fluid [2,3]. EV-associated miRNAs exhibit greater stability and diagnostic reliability for disease detection and monitoring [4]. miRNAs are involved in numerous physiological and pathological processes, and play critical roles in inflammatory responses, as well as in the development of diseases [5].

Saliva is rich in EVs and it provides a non-invasive, highly accessible source and cost-effective sampling compared to other body fluids like blood and cerebrospinal fluid. Additionally, circulating miRNAs in saliva have been successfully utilized for the early detection of head and neck cancers [6], autoimmune disorders [7], infectious diseases [8], diabetes mellitus [9], and periodontitis [10]. Furthermore, the molecular components of salivary EVs elucidates their pathogenic, diagnostic, and therapeutic roles in periodontitis [11]. For example, one study found that hsa-miR-140-5p, hsa-miR-146a-5p and hsa-miR-628-5p from salivary small EVs are associated with periodontal disease status [12].

Periodontitis is a chronic polymicrobial inflammatory disease marked by dysregulated host immune responses, which lead to the progressive destruction of connective tissue and alveolar bone, and may ultimately result in tooth loss if left untreated [13]. Periodontitis diagnosis relies on clinical parameters—including clinical attachment loss (CAL), periodontal probing depth (PPD), and bleeding on probing (BOP) and radiographic assessment of alveolar bone loss. The 2017 AAP/EFP World Workshop established a framework for classifying periodontitis into Stages I–IV based on disease severity and management complexity, with the extent of disease further categorized as localized, generalized, or molar–incisor pattern [14]. The periodontal inflamed surface area (PISA) enables a more precise assessment of periodontal disease severity and inflammatory status, and multiple studies have employed PISA to evaluate periodontal status [15,16]. It is a quantitative index that estimates the total surface area of bleeding periodontal pocket epithelium (mm^2^), calculated from PPD and BOP [17]. It provides a continuous measure of periodontal inflammatory burden, eliminating the need for summary indices, such as mean or maximum values. Chronic periodontitis affects approximately 30% of the general population. Severe generalized periodontitis occurs in 5–15% of individuals worldwide and represents a major cause of tooth loss [18,19]. Furthermore, systemic conditions can affect periodontitis, and previous studies have reported various risk factors for periodontitis, including diabetes mellitus [20], obesity [21], human immunodeficiency virus (HIV) infection or acquired immunodeficiency syndrome (AIDS) [22], and antiretroviral therapy (ART) in children with HIV [23].

ART has led to a significant reduction in opportunistic infections, decreased mortality, and improved survival and quality of life among individuals with HIV infection [24]. However, ART itself can cause adverse effects in people with HIV (PWH), negatively affecting their overall health and quality of life [23]. Children with HIV who receive ART exhibit a significantly higher prevalence of periodontal diseases (OR = 3.11, 95% CI 1.62–5.97) [23]. However, the mechanisms and roles of miRNAs in periodontitis among PWH receiving ART are little known.

We hypothesized that miRNAs from saliva-derived EVs may play roles in periodontitis in PWH receiving ART. We performed a two-phase study in periodontitis patients to test this hypothesis. In the first phase, participants were divided into two groups: individuals with periodontitis and HIV infection (case group) and individuals with periodontitis without HIV infection (control group), to identify a specific miRNA predictive of periodontitis in PWH receiving ART. In the second phase, we investigated the association between the miRNA expression and severity of periodontitis among PWH receiving ART.

## 2. Results

### 2.1. Characteristics of Salivary EVs

**Figure 1:**
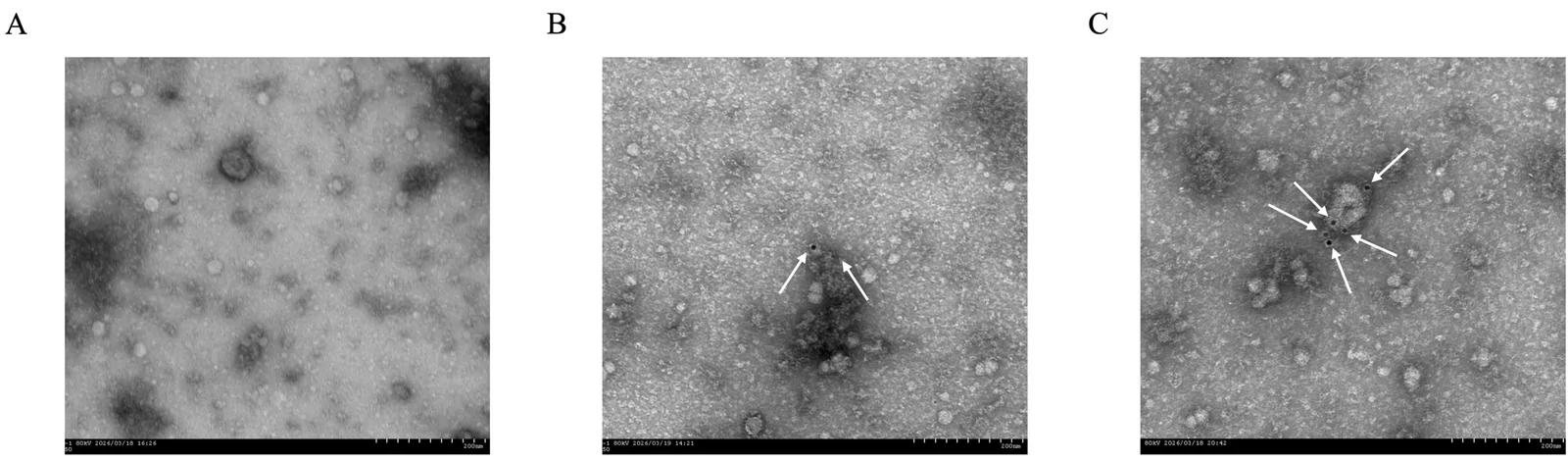
Representative images of EV characteristics after their isolation from saliva. (**A**) Representative immunogold TEM results of salivary EVs; (**B**) presence of marker CD9 (arrows) attached to the EVs identified by TEM; (**C**) presence of CD63 markers (arrows) attached to the EVs.

Transmission electron microscopy (TEM) demonstrated that the isolated salivary EVs exhibited a characteristic cup-shaped morphology (Figure 1A). Immunogold TEM confirmed the presence of EV surface markers for CD9 (Figure 1B) and CD63 (Figure 1C).

### 2.2. Phase 1 Study

#### 2.2.1. Demographic and Clinical Variables

Table 1 illustrates the characteristics of the participants in the phase 1 and phase 2 studies. Table 2 presents a comparison of the case and control groups in the phase 1 study. One patient had not been inspected for CD4 count. So, the data of CD4 count was collected from 14 patients.

#### 2.2.2. Next-Generation Sequencing (NGS) Analysis

In NGS analysis, a total of 201 miRNAs were differentially expressed in both groups. From the list, 144 miRNAs were selected from the case group by filtering the data for expression levels of zero (non-expression) in the control group. This allowed us to get the list of miRNAs from two groups. Among these 144 miRNAs, only the top miRNA (hsa-miR-324-5p) was selected on the basis of log_2_FC value (6.63) (Table S1).

### 2.3. Phase 2 Study

#### Correlation Between hsa-miR-324-5p and Clinical Variables

Table 3 shows the association between salivary miR-324-5p expression and other factors in the case group in the phase 2 study. Ten PWHs were receiving bictegravir + tenofovir alafenamide, and five PWHs were on dolutegravir + tenofovir alafenamide as the ART medication.

Reverse transcription-quantitative polymerase chain reaction (RT-qPCR) analysis revealed that salivary EV-derived hsa-miR-324-5p expression in the case group was significantly associated with the PISA (*p* = 0.028) (Table 3). Age, probing pocket depth (PPD), bleeding on probing (BOP), CD4 count, and duration of ART intake were not significantly associated with miR-324-5p expression.

### 2.4. Regulatory Network and Functional Analysis of hsa-miR-324-5p

**Figure 2:**
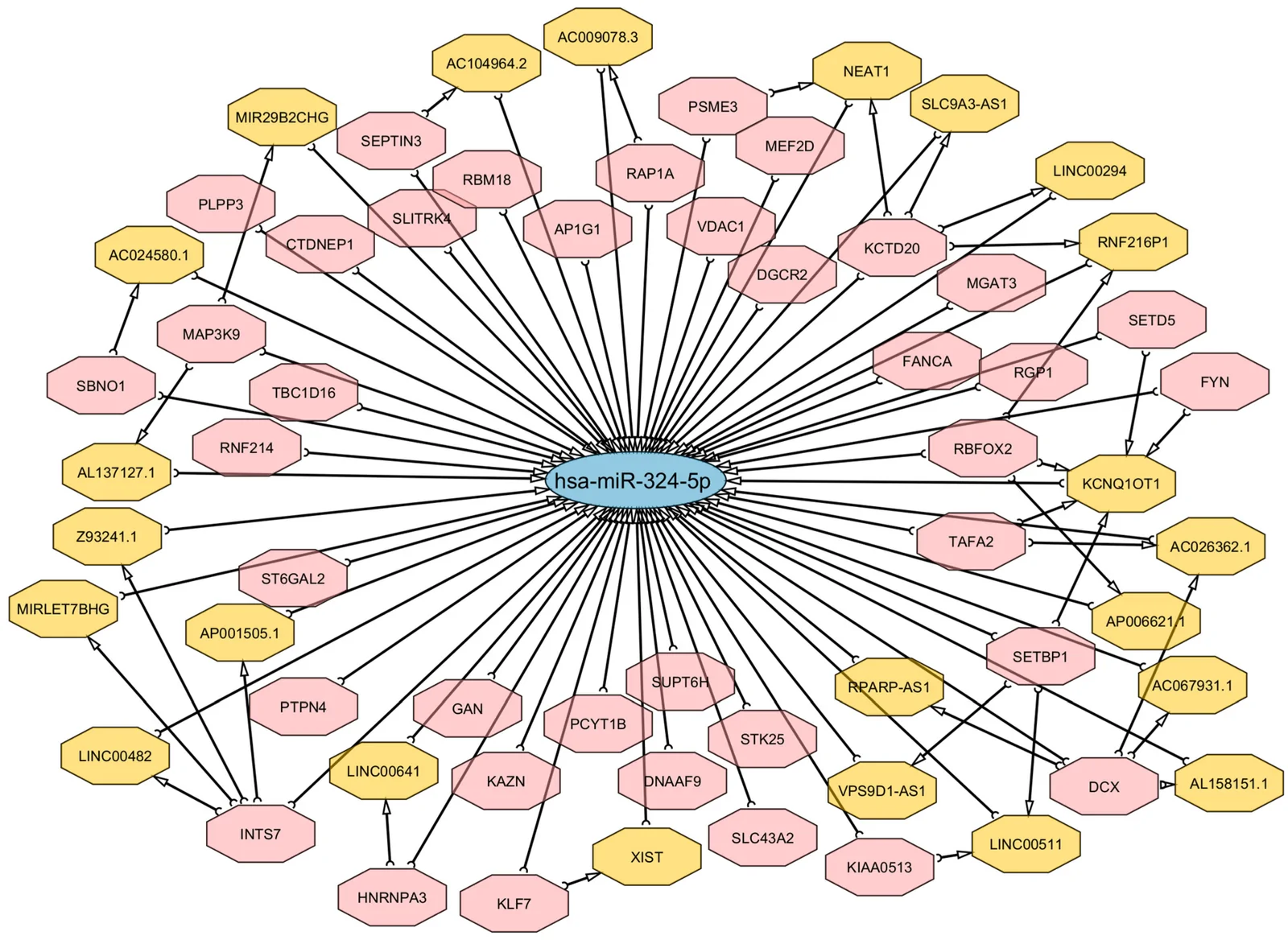
ceRNA network of hsa-miR-324-5p (blue circle) and target mRNA (pink box), and target IncRNA (light brown box).

A total of 88 mRNAs and 36 long non-coding RNAs (lncRNAs) were identified as potential targets of hsa-miR-324-5p. Based on these interactions, a competing endogenous RNA (ceRNA) regulatory network was constructed and visualized in Cytoscape (Figure 2), illustrating the complex regulatory relationships between miRNA, mRNAs, and lncRNAs.

**Figure 3:**
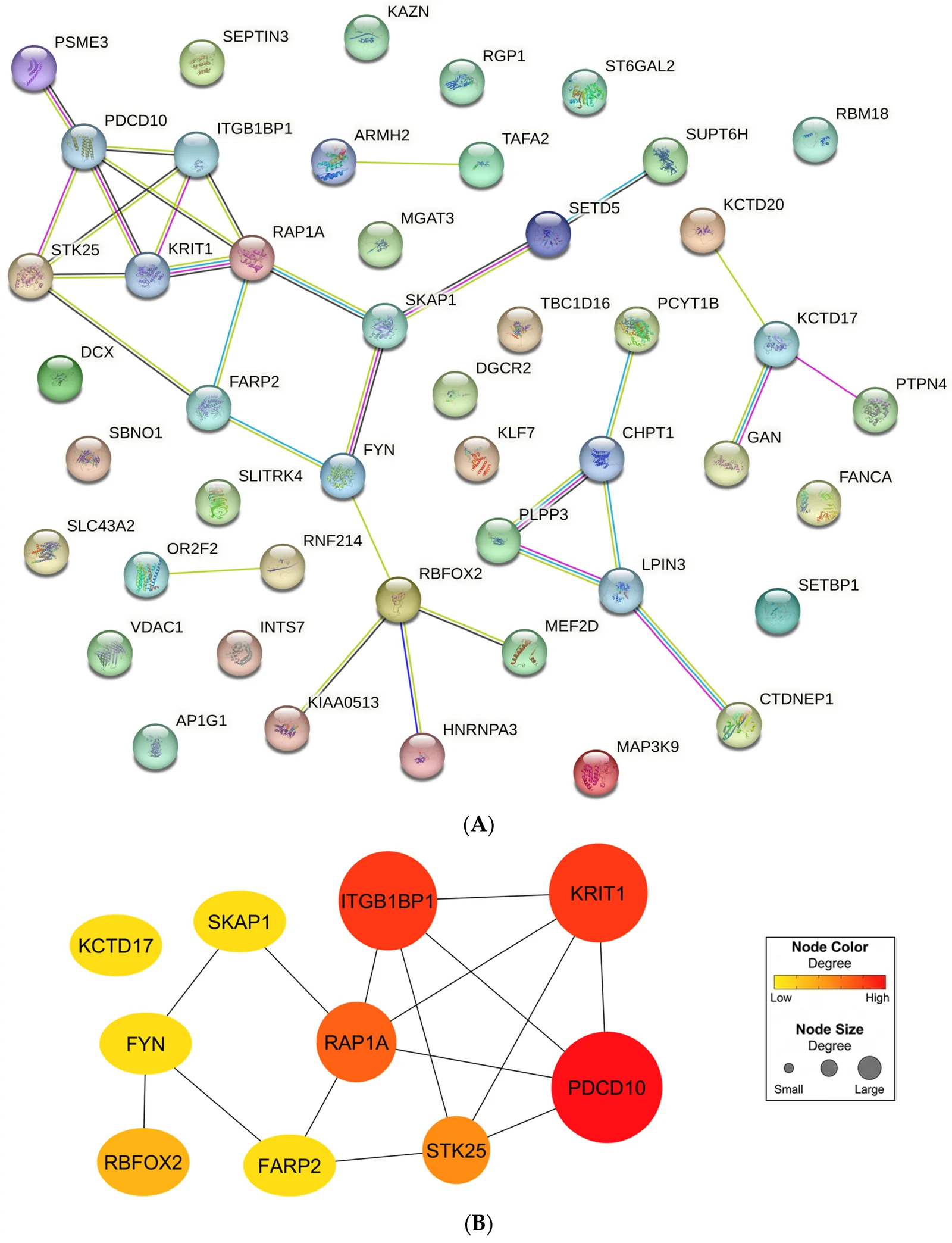
Protein–protein interaction network and potential hub genes network. (**A**) PPI network of 88 mRNAs with hsa-miR-324-5p. The colored lines indicate PPIs. Light blue indicates database-supported associations, purple indicates experimentally demonstrated associations, red indicates fusion genes, yellow-green indicates associations supported by previous studies, green indicates gene neighborhoods, blue indicates co-occurring genes, and black indicates co-expressing genes. Nodes with protein structures indicate proteins with known or predicted 3D structures. Empty nodes indicate proteins with unknown 3D structures. (**B**) The 10 predicted hub genes extracted from the PPI network.

To further characterize protein-level interactions for the 88 mRNAs of hsa-miR-324-5p, a protein–protein interaction (PPI) network was generated using the STRING database. The resulting network consisted of 47 nodes and 31 edges, indicating moderate functional connectivity between the predicted target proteins (Figure 3A). Hub gene analysis was performed using cytoHubba (version 0.1), which identified ten key hub genes within the PPI network (Figure 3B). The hub gene subnetwork contained ten nodes and fifteen edges.

**Figure 4:**
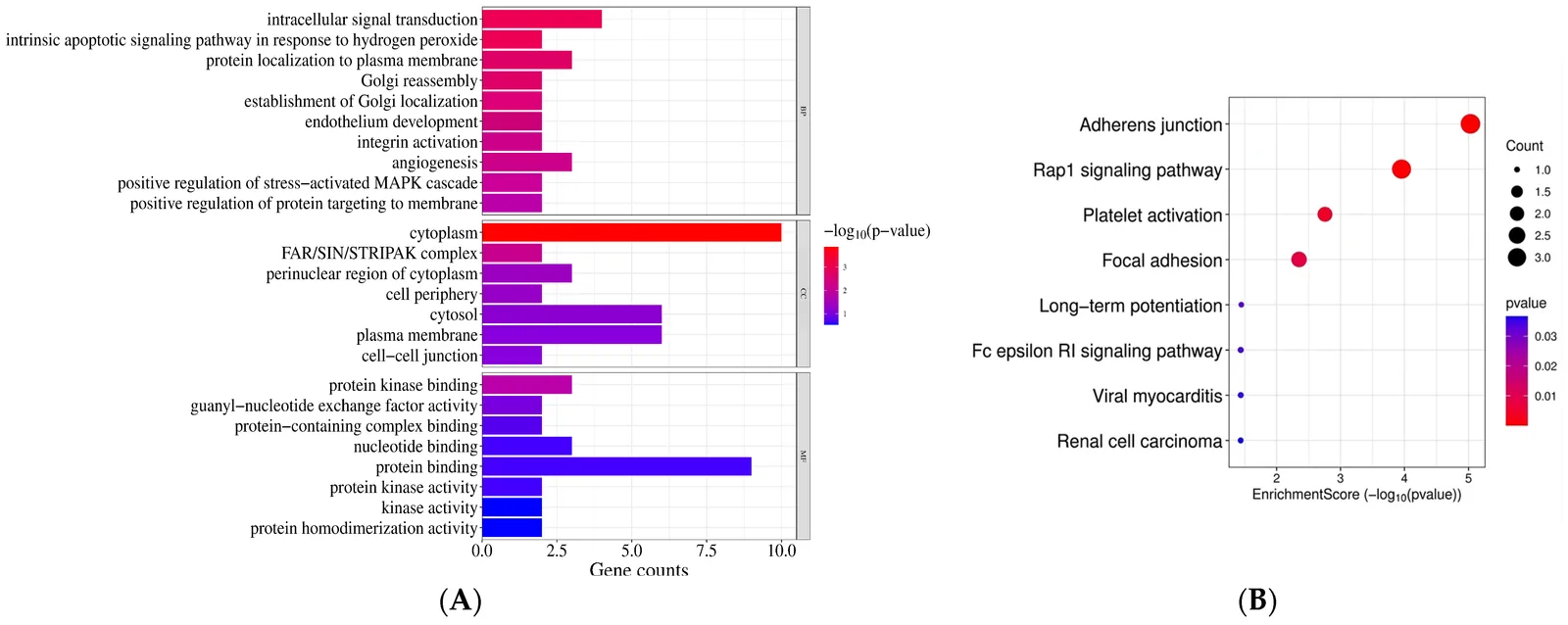
Gene Ontology (GO) and Kyoto Encyclopedia of Genes and Genomes (KEGG) functional enrichment analysis of the 10 hub genes. (**A**) Cellular components, biological processes, and molecular functions. (**B**) KEGG pathways analysis.

Gene Ontology (GO) analysis indicated that these genes are primarily localized to intracellular and cytoplasmic compartments, including the FAR/SIN/STRIPAK complex, and may significantly be involved in intracellular signal transduction, particularly protein binding and protein kinase activity (Figure 4A). These processes are closely associated with inflammatory signaling and may contribute to extracellular matrix degradation, including collagen breakdown. Kyoto Encyclopedia of Genes and Genomes (KEGG) pathway analysis further revealed significant enrichment in the adherens junction and Rap1 signaling pathway (Figure 4B).

## 3. Discussion

**Figure 5:**
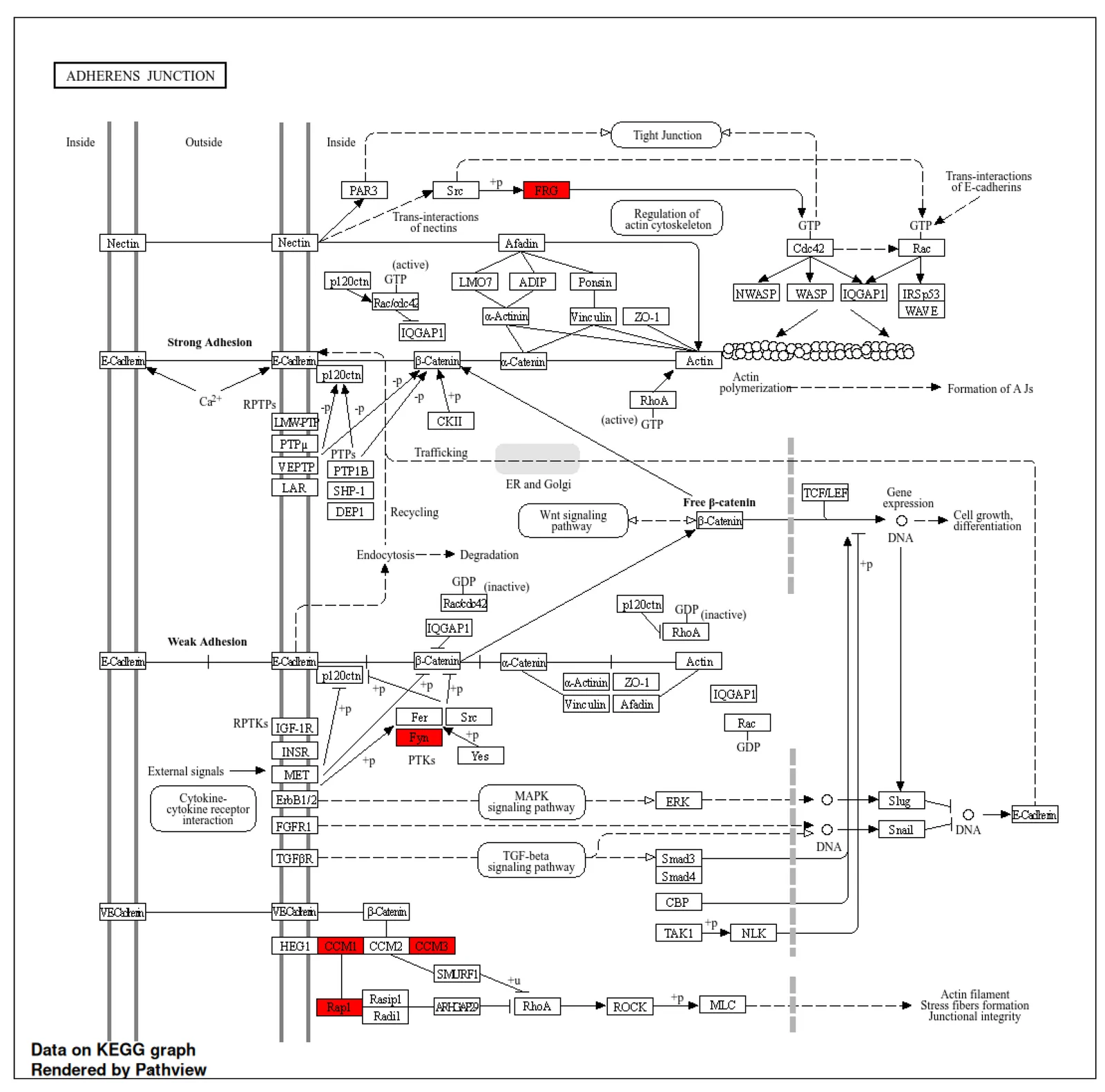
Kyoto Encyclopedia of Genes and Genomes (KEGG) pathway mapping showing interaction of genes in red color (FARP2 (FRG), FYN, KRIT1 (CCM1), PDCD10 (CCM3), RAP1A) with the adherens junction pathway.

To the best of our knowledge, this is the first study to assess a salivary EV-derived miRNA that has a role of causing periodontitis in PWH receiving ART. We hypothesize that specific salivary miRNA of PWH correlate with progression of periodontitis. In NGS analysis of miRNAs from salivary EVs, the miRNA that showed the greatest correlation with periodontitis based on log_2_FC values was selected. We also confirmed by RT-qPCR that hsa-miR-324-5p was associated with PISA in PWH. Bioinformatic analysis revealed that hsa-miR-324-5p interacts with the mRNAs encoding the 10 potential hub genes that are involved in intercellular signal transduction and the adherens junction (Figure 5).

Salivary hsa-miR-324-5p may be a potential marker reflecting the severity of periodontitis among PWH receiving ART. Exosomal miRNAs play a potential role in acute people with HIV [25]. In this study, NGS analysis was conducted to identify miRNAs from salivary-derived EVs, and hsa-miR-324-5p was identified as a candidate miRNA in the phase 1 study. In the phase 2 study, the results of RT-qPCR analysis demonstrated a correlation between hsa-miR-324-5p and PISA in the case group. In a previous study, the PISA index was used to precisely assess the severity of periodontal disease and inflammatory status [15]. The findings of this study suggested that hsa-miR-324-5p reflects the severity of periodontitis and may have potential roles in periodontitis in PWH receiving ART.

Bioinformatic analysis revealed that hsa-miR-324-5p interacts with mRNAs encoding 10 hub genes. Gene Ontology analysis demonstrated that these hub genes are enriched in intracellular signal transduction, protein kinase binding, and cytoplasmic localization. KEGG pathway analysis indicated that the identified hub genes are functionally associated with the Rap1 signaling pathway and adherens junction dynamics, both of which are essential for maintaining epithelial barrier function. Disruption of adherens junctions is widely implicated in periodontal disease, as compromised epithelial integrity facilitates bacterial invasion and amplifies local inflammatory responses [26,27].

Among the 10 hub genes, five (*FARP2*, *RAP1A*, *PDCD10*, *KRIT1*, *FYN*) are involved in the adherens junction pathway. Fyn, a member of the Src family kinase (SFKs), regulates essential cellular processes, such as proliferation, migration, differentiation, survival, B- and T-cell immunity, and microbial sensing via pattern recognition receptors [28]. An in vivo study demonstrated that *Fyn* deficiency promotes pro-inflammatory macrophage activation, which exacerbates septic acute lung injury [29]. Additionally, previous research indicates that Fyn phosphorylation in periodontal ligament stem cells is significantly enhanced by mechano-growth factor (MGF), which may facilitate the effect of mechanical stimulation on tissue repair through Fyn [30].

Four of the 10 identified hub genes (*FARP2*, *RAP1A*, *KRIT1*, *SKAP1*) are involved in the Rap1 signaling pathway. *RAP1A* encodes a protein that acts as a central regulator of cell–cell adhesion and junction stabilization. Its dysregulation is linked to increased vascular permeability and inflammatory cell infiltration. T and B cells lacking RAP1A display impaired integrin-mediated cell adhesion [31]. *KRIT1* maintains the endothelial barrier and limits inflammation. Knockdown of KRIT1 reduces the stability of adherens junction complexes and increases endothelial permeability via the Rap1 signaling pathway [32]. The RAP1A–KRIT1 axis is essential for stabilizing adherens junctions. KRIT1 mediates the ability of RAP1A to stabilize endothelial cell–cell junctions; notably, loss of KRIT1 results in the loss of β-catenin from cell–cell junctions [32]. Furthermore, loss of KRIT1 and PDCD10 increases vascular endothelial growth factor (VEGF) expression and promotes vascular inflammation [33]. A study investigating Src kinase-associated phosphoprotein 1 (SKAP1) in mice demonstrated that the T-cell immune adaptor SKAP1 regulates the induction of collagen-induced arthritis [34]. In addition, disruption or altered expression of another hub gene ICAP-1 (*ITGB1BP1*) is associated with defects in osteoblast function, impaired bone development, and dysregulation of integrin-mediated cell adhesion [35,36].

Based on the findings of previous studies [25,26,28,30,31,32,34,35], and the bioinformatic analysis conducted in this study, we postulated that hsa-miR-324-5p suppresses the expression of *KRIT1*, *RAP1A*, *ITGB1BP1*, *FYN*, and *PDCD10*, which may disrupt endothelial and epithelial barrier integrity, increase their permeability, and activate inflammatory signaling pathways. Collectively, these alterations may contribute to the pathogenesis of periodontitis or its severity in PWH receiving ART.

miR-324-5p has been demonstrated to influence multiple inflammatory responses. For instance, miR-324-5p contributes to cell proliferation and apoptosis in pancreatic cancer. Additionally, elevated miR-324-5p expression is associated with a poor prognosis in gastric cancer, and promotes tumor progression in these cells [37]. In periodontitis, the junctional epithelium acts as the primary barrier against microbial invasion. Our observations indicate that miR-324-5p plays a critical regulatory role in periodontal inflammation in PWH by modulating hub genes involved in adherens junction integrity and intracellular signaling. Taken together, these findings and those of previous studies suggest that hsa-miR-324-5p identified from saliva-derived EVs may serve as a potential marker associated with severity of periodontitis in PWH.

The present study focused on validating hsa-miR-324-5p, which was selected from the exploratory NGS analysis based on its expression profile and predicted biological relevance. However, several other differentially expressed miRNAs identified in the sequencing analysis were not further investigated. These candidate miRNAs represent valuable targets for future studies and should be validated in larger, independent cohorts to determine their association with periodontitis severity in PWH receiving ART.

This study has several limitations. First, this was a case–control study. Cohort studies and intervention studies are required to investigate the mechanisms of periodontitis in PWH on ART in greater detail. Second, the discovery phase of the miRNA analysis was based on pooled RNA samples from 12 participants per group, generating a single sequencing library for each group without biological replicates. In addition, the substantial reduction in sequencing reads after quality control may have limited the detection of low-abundance miRNAs. Therefore, differential expression analysis should therefore be interpreted as exploratory candidate identification rather than formal statistical evidence. Third, in this study, we could not identify any PWH with periodontitis who were not receiving ART. In Japan, most individuals diagnosed with HIV begin ART immediately after diagnosis. Fourth, the number of PWH participants was relatively small, which may limit the statistical power and generalizability of our findings. Furthermore, owing to the case–control design, this study identified an association between salivary miR-324-5p expression and periodontitis severity but could not establish a causal relationship. Fifth, because of the limited number of PWH in Japan, we included all eligible participants who met the inclusion criteria without excluding individuals with potential confounding factors, such as diabetes mellitus, hypertension, alcohol consumption and cigarette smoking. Adjustment of confounding variables such as smoking and diabetes by multiple regression analysis is not suitable due to the low number of study participants.

## 4. Materials and Methods

### 4.1. Study Design

This case–control study was conducted from August 2024 to April 2025 at Okayama University Hospital. Individuals with HIV infection and diagnosed with periodontitis were assigned to the case group, while HIV-negative individuals with periodontitis served as controls. Participants in the control group were matched for age, gender, and periodontal status with the case group. The study adhered to the Strengthening the Reporting of Observational Studies in Epidemiology (STROBE) guidelines [38]. We did not perform the calculation of sample size because this is a preliminary study.

### 4.2. Inclusion and Exclusion Criteria

The inclusion criteria were as follows: (1) PWH receiving ART treatment; (2) those who provided informed consent for study participation; (3) participants diagnosed with periodontitis [39]; and (4) age over 20 years. The exclusion criteria were: (1) inability to continue participation due to severe malaise; and (2) denial of consent for participation.

### 4.3. Participant Selection

PWH were recruited from the outpatient Department of Infectious Diseases at Okayama University Hospital. Control participants were recruited from outpatients at the Department of Preventive Dentistry, diagnosed with periodontitis, and going to receive supportive periodontal therapy (oral hygiene maintenance instruction, scaling and polishing). Participants with separate history of diabetes mellitus, hypertension, alcohol consumption and cigarette smoking were included in this study. There was sampling bias.

### 4.4. Study Phases

**Figure 6:**
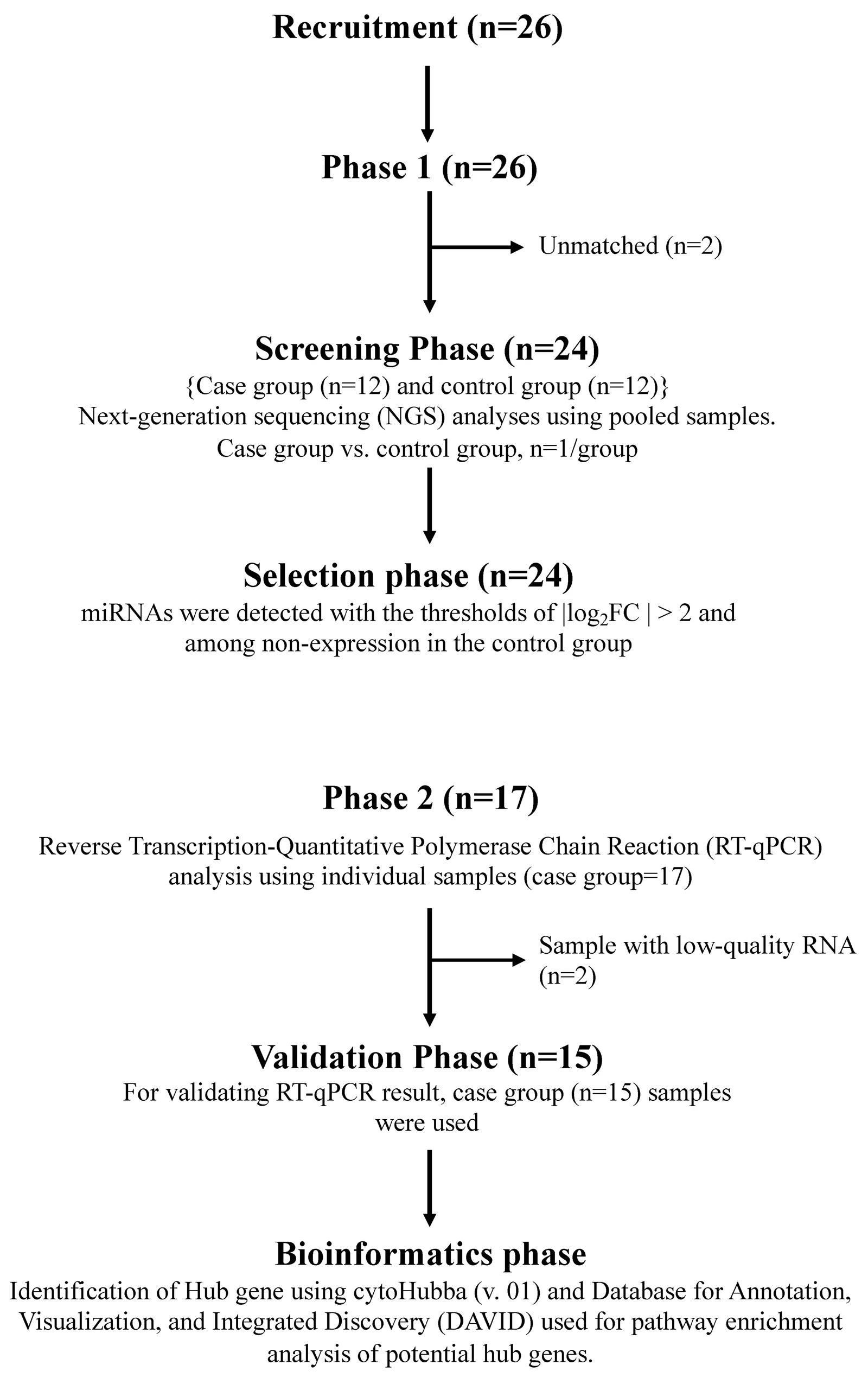
Overview of the study design.

The study was conducted in the following two phases. A flowchart of this study is presented in Figure 6.

#### 4.4.1. Phase 1 (Discovery Phase)

Oral examinations, data on clinical variables and saliva samples were obtained from matched participants in the case group and control group. NGS analysis was conducted to identify differentially expressed candidate miRNAs in the two groups.

#### 4.4.2. Phase 2 (Validation Phase)

A cohort of PWH with periodontitis was included for validation. RT-qPCR analysis was conducted to validate the expression of selected miRNA and assess it association with periodontal status.

### 4.5. Saliva Collection

Participants refrained from eating, drinking, or performing oral hygiene procedures (e.g., brushing, using mouthwash, and rinsing their mouths with water) for at least 1 h prior to sample collection. Unstimulated whole saliva was collected between 12:00 and 16:00 h before the supportive periodontal therapy. Saliva samples were collected by passive drooling into sterile 50 mL falcon tubes, avoiding coughing or forceful expectoration to prevent contamination. All samples were immediately stored at −80 °C until further analysis.

### 4.6. Oral Examination and Clinical Data

In all the study participants, comprehensive periodontal examinations were performed by a single dentist (A.Y.) to ensure consistency. Clinical parameters assessed included PPD, BOP, PISA, number of teeth present in the mouth, and plaque control record (PCR, %). PPD was measured at six sites per tooth (mesio-buccal, mid-buccal, disto-buccal, mesio-lingual, mid-lingual, and disto-lingual) using a standardized periodontal probe (CP-11 Color-Coded Probe, HuFriedy, Chicago, IL, USA). Staging of periodontitis was classified into four stages (Stages I–IV). Clinical attachment loss (CAL) was used to determine the stage of periodontitis. Stage I was defined as CAL of 1–2 mm, Stage II as CAL of 3–4 mm, Stage III as CAL ≥ 5 mm, and Stage IV as CAL ≥ 5 mm with the loss of five or more teeth due to periodontitis [39]. Additionally, salivary flow rate of all participants was measured in mL/min.

In addition to oral clinical parameters, demographic and clinical data, including age, sex, body mass index (BMI), cluster of differentiation (CD)4 and CD4/CD8 ratio, ART history (for the case group), and history of diabetes mellitus, hypertension, cigarette smoking and alcohol consumption were obtained from the patients’ medical records at Okayama University Hospital. All PWH were receiving ART.

### 4.7. EVs Isolation from Saliva

Saliva samples were centrifuged at 1200× *g* for 20 min at 4 °C to remove cellular debris and collect supernatant. From each sample, 1 mL of supernatant was used for EVs isolation. EVs were purified using magnetic bead-based separation with the MagCapture Exosome Isolation Kit PS Ver.2 (FUJIFILM Wako Pure Chemical, Osaka, Japan), according to the manufacturer’s protocol. A buffer solution was prepared and combined with exosome washing buffer, exosome elution buffer, and distilled water for preparing exosome capture-immobilized beads. A 1/500 exosome-binding enhancer was added to a 1 mL saliva sample, which was then transferred to the exosome capture-immobilized beads and incubated for 1 h using a rotator. The extracellular vesicle-binding beads were washed three times, after which the supernatant was removed, and exosome elution buffer was added to obtain purified EVs. Following isolation, the EVs were stored at −80 °C.

### 4.8. Immunogold Transmission Electron Microscopy (TEM)

The morphology and surface marker expression of saliva-derived EVs were examined using immunogold transmission electron microscopy (TEM). EV samples were adsorbed onto hydrophilized 100-mesh nickel grids and blocked with PBS containing 10% goat serum (GEMINI Bio, San Carlos, CA, USA) and 1% bovine serum albumin (BSA).

Grids were incubated with the primary antibodies CD9 and CD63 for two hours at room temperature. Following this step, the grids were incubated overnight at 4 °C. Subsequently, the grids were washed five times for 5 min each with phosphate-buffered saline (PBS) containing 0.1% BSA. The grids were then incubated for two hours with secondary antibodies (antimouse IgG/goat and anti-rabbit IgG/goat; BioLegend, San Diego, CA, USA) conjugated to 10 nm gold colloids (BBI Solutions, Crumlin, UK). The samples were washed three times for five minutes each with PBS containing 0.1% BSA, then fixed in 2% glutaraldehyde for three minutes. After rinsing with distilled water, the grids were stained with uranyl acetate for approximately 30 s, air-dried, and examined using a transmission electron microscope (H-7650; Hitachi High-Technologies, Tokyo, Japan).

### 4.9. RNA Extraction

Total RNA was extracted from purified EVs using the Norgen Exosomal RNA Isolation Kit (Norgen Biotek Corp., Thorold, ON, Canada) according to the manufacturer’s protocol. The concentration of extracted RNA was determined by measuring absorbance at 260 nm. RNA purity was evaluated using the 260/280 nm absorbance ratio with a UV spectrophotometer (Beckman Coulter, Tokyo, Japan).

### 4.10. Small RNA Library Preparation and Sequencing

Total RNA isolated from case and control samples (*n* = 12 each) were pooled for small RNA library preparation using the NEBNext Multiplex Small RNA Library Prep Set (New England Biolabs, Ipswich, MA, USA) following the manufacturer’s protocol. Briefly, 3′ and 5′ adapters were sequentially ligated to the small RNA molecules, followed by reverse transcription and PCR amplification to generate indexed cDNA libraries. The prepared libraries were purified and size-selected to enrich for small RNA fragments and subsequently subjected to high-throughput sequencing. Single-end sequencing (50 bp read length; SE50) was performed on the Illumina NovaSeq 6000 platform (San Diego, CA, USA).

### 4.11. Small RNA Raw Data Processing

Raw sequencing reads were subjected to quality assessment using FastQC (Version 0.11.7; https://www.bioinformatics.babraham.ac.uk/projects/fastqc/, accessed on 24 February 2025). A total of approximately 16.32 million reads (case group) and 14.42 million reads (control group) were obtained. Adapter sequences and low-quality bases (Phred score < 20) were removed using the sequence trimmer Trimmomatic (version 0.38). Reads shorter than 20 nucleotides after trimming were discarded to ensure high-quality downstream analysis. After filtering, approximately 1.02 million clean reads for the case group and 0.51 million clean reads for the control group were retained. The high-quality reads were aligned to the reference genome using the RNA-seq aligner STAR (version 2.7.4) with parameters optimized for small RNA mapping. The resulting alignment .bam files were used to quantify read counts mapped to annotated miRNAs using FeatureCounts (version 1.6.3), allowing multi-mapped reads.

### 4.12. Identification of Potential miRNA

Differential expression analysis of miRNAs between the two groups was performed using the edgeR (Version 3.26.8) package in R. Raw read counts obtained from small RNA sequencing were used as input for the analysis. Prior to differential expression analysis, read counts were normalized using the Trimmed Mean of M values (TMM) method to correct for differences in library size and sequencing depth among samples. The raw read counts were normalized with transcripts per million (TPM). Differential expression of miRNAs between case and control groups was determined by calculating the log_2_ fold change (log_2_FC) using the normalized read counts. miRNAs of those with non-expression in the control group were selected. Next, the log_2_ (case/control) formula was used for calculating the fold change. miRNAs were considered differentially expressed if they met the criteria of |log_2_FC| > 2.

### 4.13. RT-qPCR Validation of miRNA Expression

RT-qPCR was conducted to validate the expression levels of selected miRNA. TaqMan MicroRNA Assays (Thermo Fisher Scientific, Waltham, MA, USA) were employed for hsa-miR-324-5p (Assay ID: 002216). Small nuclear RNA U6 (Assay ID: 001973) was used as the endogenous internal control for normalization.

RT-qPCR reactions were performed using TaqMan Universal Master Mix II, no Uracil-N-glycosylase (UNG) (4440043; Thermo Fisher Scientific), in accordance with the manufacturer’s instructions through Applied Biosystems StepOnePlus™ Real-Time PCR System (Thermo Fisher Scientific, Inc.). Amplification was carried out under the following thermal cycling conditions: UNG activation at 50 °C for one cycle and one cycle enzyme activation at 95 °C for 10 min, followed by 40 cycles of denaturation at 95 °C for 15 s and annealing/extension at 60 °C for 60 s. All reactions were conducted in technical duplicates. The relative miRNA expression level of hsa-miR-324-5p was determined using the 2^(ΔΔCt)^ method, where Ct values were normalized to U6.

### 4.14. Functional Analysis of miRNA and Prediction of Target Genes

The functional roles of hsa-miR-324-5p were investigated by predicting target genes using multiple established databases, including TargetScan (v8.0) [40], miRWalk (v3.0) [41], and miRDB (http://mirdb.org) [42]. Only the overlapping target genes identified across all three databases were retained as candidate mRNAs.

Long non-coding RNAs (lncRNAs) interacting with miR-324-5p were identified using the ENCORI (https://rnasysu.com/encori/, accessed on 21 January 2026) database [43]. Predicted miRNA–mRNA and miRNA–lncRNA interactions were subsequently used to construct a competing endogenous RNA (ceRNA) regulatory network using Cytoscape (version 3.10.4).

To further investigate the interactions among the predicted target genes, a protein–protein interaction (PPI) network was generated using the Search Tool for the Retrieval of Interacting Genes/Proteins (STRING) (https://string-db.org/, accessed on 21 January 2026) [44]. Hub genes within the PPI network were identified using cytoHubba (version 0.1) [45]. Functional enrichment analysis of the identified hub genes was conducted using the Database for Annotation, Visualization, and Integrated Discovery (DAVID) (https://david.ncifcrf.gov/, accessed on 23 January 2026) [46]. Gene Ontology (GO) analysis of miRNA and their target mRNAs, and Kyoto Encyclopedia of Genes and Genomes (KEGG) pathway enrichment analysis were performed to elucidate the potential biological processes and signaling pathways associated with hsa-miR-324-5p.

### 4.15. Statistical Analysis

All statistical analyses were conducted using IBM SPSS Statistics version 25 (IBM Corp., Chicago, IL, USA). Participant characteristics and oral health variables are presented as the mean ± standard deviation (SD). Between-group comparisons utilized the independent samples *t*-test. Spearman’s rank correlation analysis was used to evaluate associations between miRNA expression levels and clinical variables within the case group. A two-tailed *p*-value less than 0.05 was considered statistically significant for all analyses.

## 5. Conclusions

This study demonstrated that hsa-miR-324-5p identified from saliva-derived EVs was associated with PISA in PWH receiving ART. Bioinformatic analysis predicted that the target genes of hsa-miR-324-5p may be involved in protein kinase binding, the Rap1 signaling pathway, and adherens junction dynamics. These findings provide hypotheses regarding the potential biological functions of hsa-miR-324-5p in periodontal disease but do not establish a causal role in the progression of periodontitis. Further experimental studies may be required to validate these predicted target genes and clarify the underlying molecular mechanisms.

## Figures and Tables

**Table 1:** Characteristics of the participants.

Variable	Phase 1 (*n* = 24)	Phase 2 (*n* = 15)
Age (years)	62.6 ± 16.3 *	58.2 ± 18.1
Female	0	1 (6.6) ^†^
Number of teeth present	21.3 ± 8.0	21.5 ± 8.1
Mean PPD (mm)	2.6 ± 0.4	2.6 ± 0.4
BOP (%)	21.5 ± 18.2	26.1 ± 20.7
BMI (kg/m^2^)	23.5 ± 3.2	24.5 ± 3.2
Plaque control record (%)	50.3 ± 28.6	50.7 ± 30.3
Periodontal staging (III/IV)	20 (83.3)	11 (73.3)
Salivary flow rate (mL/min)	0.5 ± 0.3	0.6 ± 0.5
CD4 count (10^3^/µL)	0.5 ± 0.2 ^¶^	0.5 ± 0.2
CD4/CD8 ratio	1.0 ± 0.5 ^¶^	1.0 ± 0.4
Duration of ART (years)	13.5 ± 6.4 ^¶^	12.9 ± 5.7
Current smoker	6 (25.0)	4 (26.6)
Alcohol consumption (+)	9 (37.5)	4 (26.6)
Diabetes mellitus	5 (20.1)	3 (20.0)
Hypertension	10 (41.6)	7 (46.7)

PPD, probing pocket depth; BOP, bleeding on probing; BMI, body mass index. * Mean ± SD; ^†^ Number (%); ^¶^ PWH participants.

**Table 2:** Comparison of case and control groups in phase 1.

Variable	Case Group (*n* = 12)	Control Group (*n* = 12)	95% Confidence Interval	*p*-Value ^†^
Age (years)	64.3 ± 15.3 *	61.0 ± 17.8	−10.784–17.284	0.636
Number of teeth present	18.8 ± 8.1	23.7 ± 7.3	−11.392–1.726	0.141
Mean PPD (mm)	2.7 ± 0.5	2.5 ± 0.4	−0.191–0.552	0.325
BOP (%)	25.0 ± 21.4	18.0 ± 14.5	−8.482–22.482	0.359
PISA (mm^2^)	251.3 ± 247.8	249.9 ± 232.8	−202.181–204.914	0.989
BMI (kg/m^2^)	24.7 ± 3.6	22.3 ± 2.3	−0.187–4.914	0.068
Plaque control record (%)	53.9 ± 33.2	46.7 ± 23.9	−17.270–31.770	0.546
Salivary flow rate (mL/min)	0.5 ± 0.4	0.5 ± 0.2	−0.258–0.307	0.859

PPD, probing pocket depth; BOP, bleeding on probing; PISA, periodontal inflamed surface area; BMI, body mass index. * Mean ± SD; ^†^ *t*-test (Significance level for multiple testing: *p* < 0.006).

**Table 3:** Association between salivary miR-324-5p expression and other factors from the case group in the phase 2 study.

Variable	Cases (*n* = 15)	
	Spearman’s Correlation Coefficient	*p*-Value ^¶^
Age	0.059	0.834
BOP	0.262	0.346
PISA	0.564	0.028 *
PPD	0.368	0.177
CD4 count	−0.121	0.681
Duration of ART intake	0.271	0.328

BOP, bleeding on probing; PISA, periodontal inflamed surface area; PPD, probing pocket depth. ^¶^ Spearman’s rank correlation analysis, * *p* < 0.05; significant.

## Data Availability

The original contributions presented in this study are included in the article/Supplementary Material. Further inquiries can be directed to the corresponding author.

## Supplementary Materials

The following supporting information can be downloaded at https://www.mdpi.com/article/10.3390/ijms27188117/s1.

## Abbreviations

The following abbreviations are used in this manuscript:

ART	Antiretroviral therapy
BOP	Bleeding on probing
BMI	Body mass index
CAL	Clinical attachment loss
ceRNA	Competing endogenous RNA
EVs	Extracellular vesicles
GO	Gene Ontology
HIV	Human immunodeficiency virus
lncRNA	Long non-coding RNAs
KEGG	Kyoto Encyclopedia of Genes and Genomes
NGS	Next-generation sequencing
PPD	Probing pocket depth
PISA	Periodontal inflamed surface area
PWH	People with HIV
TEM	Transmission electron microscopy
