# Supplementary material for: Salivary miR-324-5p Is Associated with Periodontal Inflamed Surface Area in People Living with HIV Receiving Antiretroviral Therapy: A Case–Control Study
Source: Int J Mol Sci. 2026 Sep 12;27(18):8117. doi: 10.3390/ijms27188117 (PMC13607824; doi:10.3390/ijms27188117)
Supplement: Supplementary file 1 [file ijms-27-08117-s001.zip › ijms-4403579-supplementary.pdf]

Table S1. List of miRNAs from Next-generation sequencing result in the Phase 1

miRNA_id	miRNA name	Case raw expression	logFC (case/control)
MIMAT0000761	hsa-miR-324-5p	61.24872	6.643151
MIMAT0022695	hsa-miR-212-5p	36.02866	5.887685
MIMAT0027632	hsa-miR-6866-5p	32.42579	5.738387
MIMAT0019926	hsa-miR-4772-5p	28.82293	5.571836
MIMAT0041629	hsa-miR-10399-5p	28.82293	5.571836
MIMAT0002874	hsa-miR-503-5p	25.22006	5.383517
MIMAT0005889	hsa-miR-548l	25.22006	5.383517
MIMAT0010251	hsa-miR-449c-5p	25.22006	5.383517
MIMAT0011158	hsa-miR-2115-5p	25.22006	5.383517
MIMAT0004610	hsa-miR-150-3p	21.6172	5.166873
MIMAT0004766	hsa-miR-146b-3p	21.6172	5.166873
MIMAT0005933	hsa-miR-1277-3p	21.6172	5.166873
MIMAT0019738	hsa-miR-4664-3p	21.6172	5.166873
MIMAT0025477	hsa-miR-6510-3p	21.6172	5.166873
MIMAT0002835	hsa-miR-526b-5p	18.01433	4.911849
MIMAT0004814	hsa-miR-654-3p	18.01433	4.911849
MIMAT0009451	hsa-miR-1976	18.01433	4.911849
MIMAT0015012	hsa-miR-3143	18.01433	4.911849
MIMAT0000269	hsa-miR-212-3p	14.41146	4.601852
MIMAT0003214	hsa-miR-551a	14.41146	4.601852
MIMAT0003298	hsa-miR-629-3p	14.41146	4.601852
MIMAT0003332	hsa-miR-656-3p	14.41146	4.601852
MIMAT0004498	hsa-miR-25-5p	14.41146	4.601852
MIMAT0004801	hsa-miR-590-3p	14.41146	4.601852
MIMAT0005572	hsa-miR-1225-5p	14.41146	4.601852
MIMAT0025852	hsa-miR-6721-5p	14.41146	4.601852
MIMAT0027495	hsa-miR-6797-3p	14.41146	4.601852
MIMAT0027513	hsa-miR-6806-3p	14.41146	4.601852
MIMAT0030417	hsa-miR-6516-5p	14.41146	4.601852
MIMAT0031074	hsa-miR-450a-2-3p	14.41146	4.601852
MIMAT0003244	hsa-miR-579-3p	10.8086	4.206484
MIMAT0004507	hsa-miR-92a-1-5p	10.8086	4.206484
MIMAT0004569	hsa-miR-222-5p	10.8086	4.206484
MIMAT0004585	hsa-let-7i-3p	10.8086	4.206484
MIMAT0004589	hsa-miR-30b-3p	10.8086	4.206484
MIMAT0004594	hsa-miR-132-5p	10.8086	4.206484
MIMAT0004902	hsa-miR-891a-5p	10.8086	4.206484
MIMAT0009198	hsa-miR-224-3p	10.8086	4.206484
MIMAT0015038	hsa-miR-3164	10.8086	4.206484
MIMAT0017981	hsa-miR-3605-5p	10.8086	4.206484

MIMAT0017993	hsa-miR-3614-3p	10.8086	4.206484
MIMAT0019700	hsa-miR-4640-3p	10.8086	4.206484
MIMAT0022717	hsa-miR-873-3p	10.8086	4.206484
MIMAT0025450	hsa-miR-6499-5p	10.8086	4.206484
MIMAT0026917	hsa-miR-1910-3p	10.8086	4.206484
MIMAT0028221	hsa-miR-7155-3p	9.007165	3.958995
MIMAT0000071	hsa-miR-17-3p	7.205732	3.660074
MIMAT0000072	hsa-miR-18a-5p	7.205732	3.660074
MIMAT0003220	hsa-miR-556-5p	7.205732	3.660074
MIMAT0003260	hsa-miR-592	7.205732	3.660074
MIMAT0003333	hsa-miR-549a-3p	7.205732	3.660074
MIMAT0003888	hsa-miR-766-3p	7.205732	3.660074
MIMAT0004511	hsa-miR-99a-3p	7.205732	3.660074
MIMAT0004793	hsa-miR-556-3p	7.205732	3.660074
MIMAT0004911	hsa-miR-874-3p	7.205732	3.660074
MIMAT0004929	hsa-miR-190b-5p	7.205732	3.660074
MIMAT0015053	hsa-miR-3176	7.205732	3.660074
MIMAT0015068	hsa-miR-3186-3p	7.205732	3.660074
MIMAT0017982	hsa-miR-3605-3p	7.205732	3.660074
MIMAT0018205	hsa-miR-3928-3p	7.205732	3.660074
MIMAT0019218	hsa-miR-3194-3p	7.205732	3.660074
MIMAT0019971	hsa-miR-4796-3p	7.205732	3.660074
MIMAT0022711	hsa-miR-660-3p	7.205732	3.660074
MIMAT0027454	hsa-miR-6777-5p	7.205732	3.660074
MIMAT0031179	hsa-miR-7976	7.205732	3.660074
MIMAT0037328	hsa-miR-549a-5p	7.205732	3.660074
MIMAT0000276	hsa-miR-219a-5p	3.602866	2.76991
MIMAT0000685	hsa-miR-34b-5p	3.602866	2.76991
MIMAT0000720	hsa-miR-376c-3p	3.602866	2.76991
MIMAT0000760	hsa-miR-331-3p	3.602866	2.76991
MIMAT0001618	hsa-miR-191-3p	3.602866	2.76991
MIMAT0001636	hsa-miR-452-3p	3.602866	2.76991
MIMAT0002820	hsa-miR-497-5p	3.602866	2.76991
MIMAT0003180	hsa-miR-487b-3p	3.602866	2.76991
MIMAT0004513	hsa-miR-101-5p	3.602866	2.76991
MIMAT0004514	hsa-miR-29b-1-5p	3.602866	2.76991
MIMAT0004587	hsa-miR-23b-5p	3.602866	2.76991
MIMAT0004681	hsa-miR-26a-2-3p	3.602866	2.76991
MIMAT0004683	hsa-miR-362-3p	3.602866	2.76991
MIMAT0004907	hsa-miR-892a	3.602866	2.76991
MIMAT0004975	hsa-miR-509-3-5p	3.602866	2.76991
MIMAT0005593	hsa-miR-1238-3p	3.602866	2.76991
MIMAT0005880	hsa-miR-1290	3.602866	2.76991
MIMAT0005883	hsa-miR-1293	3.602866	2.76991

MIMAT0005924	hsa-miR-1270	3.602866	2.76991
MIMAT0014983	hsa-miR-3121-3p	3.602866	2.76991
MIMAT0015050	hsa-miR-323b-3p	3.602866	2.76991
MIMAT0015051	hsa-miR-3174	3.602866	2.76991
MIMAT0018080	hsa-miR-3659	3.602866	2.76991
MIMAT0018358	hsa-miR-3942-5p	3.602866	2.76991
MIMAT0018927	hsa-miR-378e	3.602866	2.76991
MIMAT0018933	hsa-miR-4420	3.602866	2.76991
MIMAT0019012	hsa-miR-3155b	3.602866	2.76991
MIMAT0019040	hsa-miR-4504	3.602866	2.76991
MIMAT0019048	hsa-miR-4511	3.602866	2.76991
MIMAT0019058	hsa-miR-4521	3.602866	2.76991
MIMAT0019232	hsa-miR-4423-5p	3.602866	2.76991
MIMAT0019233	hsa-miR-4446-5p	3.602866	2.76991
MIMAT0019739	hsa-miR-4665-5p	3.602866	2.76991
MIMAT0019807	hsa-miR-4707-5p	3.602866	2.76991
MIMAT0019841	hsa-miR-4724-5p	3.602866	2.76991
MIMAT0019911	hsa-miR-4762-3p	3.602866	2.76991
MIMAT0019943	hsa-miR-4781-3p	3.602866	2.76991
MIMAT0020958	hsa-miR-4482-3p	3.602866	2.76991
MIMAT0022266	hsa-miR-548ar-3p	3.602866	2.76991
MIMAT0022287	hsa-miR-5586-5p	3.602866	2.76991
MIMAT0022475	hsa-miR-5685	3.602866	2.76991
MIMAT0022708	hsa-miR-584-3p	3.602866	2.76991
MIMAT0022714	hsa-miR-766-5p	3.602866	2.76991
MIMAT0022726	hsa-miR-1306-5p	3.602866	2.76991
MIMAT0022842	hsa-miR-98-3p	3.602866	2.76991
MIMAT0024598	hsa-miR-6125	3.602866	2.76991
MIMAT0025463	hsa-miR-6503-3p	3.602866	2.76991
MIMAT0025485	hsa-miR-6514-3p	3.602866	2.76991
MIMAT0026477	hsa-miR-128-1-5p	3.602866	2.76991
MIMAT0026479	hsa-miR-152-5p	3.602866	2.76991
MIMAT0026765	hsa-miR-1537-5p	3.602866	2.76991
MIMAT0027475	hsa-miR-6787-3p	3.602866	2.76991
MIMAT0027487	hsa-miR-6793-3p	3.602866	2.76991
MIMAT0027536	hsa-miR-6818-5p	3.602866	2.76991
MIMAT0027537	hsa-miR-6818-3p	3.602866	2.76991
MIMAT0030418	hsa-miR-6516-3p	3.602866	2.76991
MIMAT0037331	hsa-miR-147b-5p	3.602866	2.76991
MIMAT0041615	hsa-miR-10392-5p	3.602866	2.76991
MIMAT0041993	hsa-miR-9983-3p	3.602866	2.76991
MIMAT0006764	hsa-miR-320d	2.990379	2.543779
MIMAT0003257	hsa-miR-550a-3p	2.41392	2.292734
MIMAT0019021	hsa-miR-4487	2.41392	2.292734

MIMAT0005892	hsa-miR-1304-5p	2.16172	2.167484

logFC: log2(Fold Change)
